# Efficacy and safety of fecal microbiota transplant in irritable bowel syndrome: An update based on meta‐analysis of randomized control trials

**DOI:** 10.1002/hsr2.814

**Published:** 2022-09-12

**Authors:** Yomna Ali Abdelghafar, Yossef Hassan AbdelQadir, Karam R. Motawea, Sara Amr Nasr, Hoda Aly Mohamed Omran, Mohamed Mohamed Belal, Mohamed Mahdy Elhashash, Ahmed Alaa AbdelAzim, Jaffer Shah

**Affiliations:** ^1^ Faculty of Medicine Alexandria University Alexandria Egypt; ^2^ Faculty of Medicine Beni Suef University Beni Suef Egypt; ^3^ New York State Department of Health New York NY USA

**Keywords:** constipation, fecal microbiota transfer, irritable bowel syndrome, quality of life

## Abstract

**Background and Aims:**

Fecal microbiota transfer (FMT) is a potential treatment for irritable bowel syndrome (IBS). Several randomized trials have tested FMT effects using different routes of administration, doses, and sample sizes. We aim to assess the overall efficacy of FMT for IBS patients and the safety of the intervention.

**Methods:**

We systematically searched four databases for randomized control trials that studied the efficacy and safety of FMT in IBS patients.

**Results:**

We included 8 randomized trials (472 patients) that compared FMT with placebo in IBS patients. Pooled results showed no statistically significant difference between FMT and control groups in the overall change in IBS symptom severity (IBS‐SSS) at 1 month (*p* = 0.94), 3/4 months (*p* = 0.82), and at the end of trials (*p* = 0.67). No significant difference in the total number of respondents between the FMT and control groups (risk ratios = 1.84, [95% confidence interval (CI) = 0.82–2.65], *p* = 0.19). Although the oral route of administration showed a significant difference in the number of respondents (*p* = 0.004), there was no statistically significant difference in the IBS‐SSS when subgrouping the oral route of administration (mean difference = 47.57, [95% CI = −8.74–103.87], *p* = 0.10).

**Conclusion:**

FMT is not an effective treatment to relieve all the symptoms of IBS. Even in the groups that showed relatively significant improvement after FMT, the effect was proven to wear off over time and the re‐administration carries a low success rate. Future research should consider different bacterial‐based interventions such as probiotics or specific antibiotics.

## 1 INTRODUCTION

Irritable bowel syndrome (IBS) is a chronic multifactorial functional gastrointestinal disorder, that develops in the middle or lower parts of the gastrointestinal tract (GIT).^1^, ^2^ It is a symptom‐based disorder that is currently clinically diagnosed by Rome criteria.^3^, ^4^

Rome III and Rome IV criteria resulted in more specific diagnosis and lower prevalence rates of IBS.^5^, ^6^ Rome IV is the last update that was released in May 2016, one of the main modifications to the Rome III criteria is that discomfort is no longer accounted for and abdominal pain is now mandatory for diagnosis, also symptom frequency to be at least once per week.^6^ IBS presents with many symptoms that include abdominal distention, bloating, and pain, as well as altered bowel habits.^3^, ^7^ According to the symptom presentation, IBS is classified into three subtypes; IBS with diarrhea (IBS‐D), IBS with constipation (IBS‐C), and IBS with mixed bowel pattern (IBS‐M).^8^

Most recent studies suggest that the worldwide prevalence of IBS currently ranges between 4% and 10%, with the lowest prevalence rates in Singapore, and the highest prevalence rates in Egypt. It is also shown that the prevalence of IBS is higher in women than in men. Regarding age, studies have shown that IBS is more common among adults, and as age increases the prevalence of IBS decreases.^5^, ^6^, ^9^, ^10^ IBS patients are more likely to suffer from depression and lower quality of life (QOL), the incidence of depression co‐occurrence in IBS patients is estimated to be between 44% and 84%.^11^

Although the exact etiology of IBS is still unknown, studies suggest that multiple factors including inflammatory agents, visceral hypersensitivity, genetic factors, disorders in gut–brain interaction, and psychosocial stress, all contribute to the pathogenesis of IBS.^7^, ^8^, ^9^ Consequently, there is an imbalance in the gut microbiota, which is known as dysbiosis, which results in a disturbance of the integrity of the mucosal epithelium as well as GIT motility.^12^, ^13^

Recent research studies on gut microbiome‐focused treatment for IBS explore the manipulation of gut microbiota by prebiotics, probiotics, antibiotics, dietary changes, and fecal microbiota transfer (FMT).^14^ In this review, we focus on FMT. FMT is a novel treatment to restore the balance of gut microbiota through the transfer of fecal microbiota of a healthy donor into the patient's GIT via either oral capsules, nasojejunal, or endoscope.^15^ It has proved efficacy in the treatment of many GIT disorders, mainly recurrent clostridium difficile infection, in addition to inflammatory bowel disease, hepatic encephalopathy, chronic constipation, and colorectal cancer with mild and self‐limited adverse effects.^16^ Other extradigestive clinical implications for FMT such as diabetes and obesity are showing promising results for future application.^17^ Although it is a cost‐effective and readily available treatment option,^18^ previously published clinical trials showed conflicting results in symptoms improvement in IBS patients and improving QOL.^2^, ^19^

There was a noticeable difference among the clinical trials in the outcome measurement, patient baseline characteristics, and the dose, preparation, and route of administration of FMT. So the aim of this meta‐analysis is to compare the efficacy of FMT with placebo through pooling the improvement in the symptom severity score (SSS) and QOL. We would also assess the safety of the procedure and if there are any associated serious adverse effects. Our study also aims to provide a better quality of evidence from the previous meta‐analyses by including only RCTs and excluding nonpeer‐reviewed reports.

## 2 MATERIALS AND METHODS

The guidelines of the Cochrane handbook of systematic reviews were followed during the conduction of this review.^20^ In addition to the regulations of preferred reporting items of systematic reviews and meta‐analysis (The PRISMA 2020 update).^21^, ^22^

### 2.1 Search strategy

We used MeSH terms to form the following search strategy ((“irritable bowel syndrome”) OR (“irritable” AND “bowel” AND “syndrome”) OR (“IBS”)) AND ((“fecal microbiota transplantation”) OR (“fecal microbiota transplant”) OR (“faecal microbiota transplantation”) OR (“faecal microbiota transplant”) OR (“feacal” AND “microbiota” AND “transplant”) OR (“fecal” AND “microbiota” AND “transplant”) OR (“FMT”)) to search four databases: PubMed, SCOPUS (Title and abstract search for terms), Cochrane library, Web Of Science on February 2021 and updated our search in October 2021, for a further check, two authors performed a manual search by screening the references of the included studies.

### 2.2 Study selection

We included randomized controlled trials comparing the fecal microbiota transplant in IBS patients diagnosed using either Rome III or IV criteria with autologous transfer or placebo group. The main outcome was the change from baseline using the IBS‐SSS scale at different time points. Our PICO criteria were:

Population: Patients with IBS.

Intervention: Fecal microbiota transplant by any route of administration and any dosage.

Comparison: Control group or autologous transfer.

Outcome: Change in IBS symptoms severity and disease control, also the safety and side effects of the intervention.

We excluded case reports, conference abstracts, and single‐arm trials. We have gone through two steps to select the eligible studies, (1) title and abstract screening and (2) full‐text screening; authors were grouped into two groups and each group performed the screening and data collection separately. The leader author resolved the disputes and compared the results from the two groups. The first and second authors were primarily responsible for data analysis and writing.

### 2.3 Data extraction

We extracted the data from the included studies in two Excel sheets, in the first one, two authors extracted baseline characteristics of the eligible patients: age, BMI, sex, years since the diagnosis, type of IBS, and so forth, and the other contained outcomes measurement, we divided the main outcomes into (a) primary outcomes: Change from baseline in IBS symptom severity score at 1, 3–4 months, and at the time of last assessment (mean/standard deviation [SD]); total number of patients who achieved 50 or more points decrease in IBS‐SSS; (b) secondary outcomes: QOL score (mean/SD); adverse events such as nausea, abdominal pain, diarrhea, constipation, and bloating. And after finishing the task every two authors revised the other two authors' work; S. A. N. and Y. H. A. revised the entire work.

### 2.4 Risk of bias assessment

We used the Cochrane tool to assess the risk of bias in randomized trials (ROB 1), as described in Chapter 8.5. of the Cochrane book depending on the following items: random sequence generation, allocation concealment, blinding of participants and personnel, blinding of outcome assessment, incomplete outcome data, selective reporting and other bias (missing protocol or funding issues would be considered as a source of risk), each item was graded as high risk, low risk, or unclear risk of bias.

### 2.5 Data analysis

We used the Review Manager Software version 5.3 to perform the meta‐analysis; the continuous outcomes were measured as mean difference (MD) and SD, and the dichotomous outcomes as risk ratios (RR) with a 95% confidence interval (CI). In case of heterogeneity (*χ* ^2^ *p* < 0.1), a random effect model was adopted, otherwise, a fixed‐effect model was employed, and we used “take one out” method to resolve the heterogeneity, in general; the results were considered significant if the *p*‐value was less than 0.05.

## 3 RESULTS

### 3.1 Literature search

**Figure 1:**
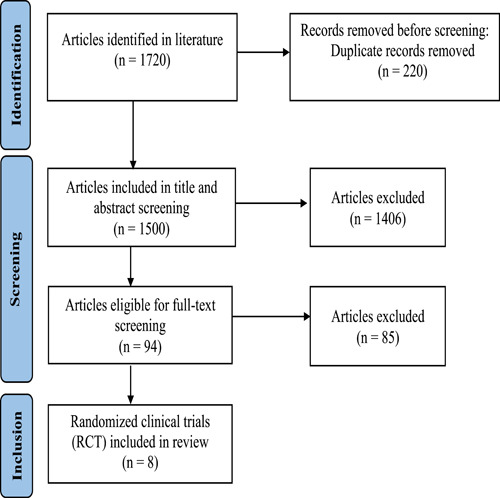
PRISMA flow chart

The literature search retrieved 1490 citations after duplicates removal. After title and abstract screening, 94 articles were retrieved for further evaluation (full‐text screening). Eight randomized trials were finally included. No other papers were found after the screening of the references of included trials and finally, 8 studies with 472 patients were included in data extraction (see PRISMA flow diagram; Figure 1).

### 3.2 Characteristics of the included studies

Change from baseline (IBS‐SSS), change from baseline after 1 month (IBS‐SSS), change from baseline after 3/4 months (IBS‐SSS), the number of patients who achieved more than or equal to 50 points decrease in IBS‐SSS score, change from baseline (QOL score), and change from baseline after 1 month (QOL score) outcomes were reported in 5, 3, 2, 4, 4, and 2 studies, respectively. Nausea, abdominal pain, diarrhea, constipation, and bloating adverse events were reported in 5, 5, 5, 3, and 4 studies, respectively. The total number of patients included in the meta‐analysis in the FMT group is 288 patients, and the total number of patients in the control group is 184 patients. Patients' baseline data and a summary of the included studies are presented in Table 1.

**Table 1:** Showing baseline characteristics and summary of the included trials.

Study ID, Year	Study Design	Study Site	Sample Size	Diagnostic Criteria Used	Study Groups	Number of Patients	Mean age (SD)
Holvoet et al., 2021^23^	Placebo‐Controlled, double‐blinded RCT	Belgium	62	Rome III	FMT	43	40(8.5)
					Placebo	19	38.25(11.25)
Lahtinen et al., 2020^24^	Randomized clinical trial	Finland	49	Rome IV	FMT	23	47.3(16.8)
					Placebo	26	46.3(14.3)
El‐Salhy et al., 2020^2^	Placebo‐Controlled, double‐blinded RCT	Norway	163	Rome III	FMT	54	39.2(12.4)
					FMT	55	39.3(13.2)
					Placebo	55	41.2(13.7)
Johnsen et al., 2020^25^	Placebo‐Controlled, double‐blinded RCT	Norway	83	Rome III	FMT	55	43.67(15.56)
					Placebo	28	45.3(17.03)
Halkjær et al., 2019^19^	Placebo‐Controlled, double‐blinded RCT	Denmark	51	Rome III	FMT	25	37.28(12.48)
					Placebo	26	35.54(10.58)
Holster et al., 2019^26^	Randomized Controlled Trial	Sweden	16	Rome III	FMT	8	36.67(16.3)
					Placebo	8	38.33(7.41)
Aroniadis et al., 2019^8^	Placebo‐Controlled, double‐blinded RCT	USA	48	Rome III	FMT	25	36(15.56)
					Placebo	23	39.33(14.81)
Johnsen et al., 2018^27^	Placebo‐Controlled, double‐blinded RCT	Norway	83	Rome III	FMT	55	43.67(15.56)
					Placebo	28	45.3(17.03)

Study ID, Year	Male n (%)	Dose	Route of Adminstration	Mean Years with IBS	IBS Subtypes	Total Follow‐up Time	Primary endpoint to define improvement	Outcomes
Holvoet et al., 2021^23^	13 (31%)	Single 300 ml dose consisting of 30 gm freshly donated feces	Nasojejunal	10		12 months	Relief of general IBS symptoms and abdominal bloating at 12 weeks	More patients in the active treatment group showed improvement in the general IBS symptoms than the placebo‐treated group.
	11 (59%)	Single 300 ml dose consisting of 30 gm patients' own feces		10.13				
Lahtinen et al., 2020^24^	11 (48%)	Single dose of 30 gm fecal suspension from a single universal donor	Gastroscope		IBS‐D: 9, IBS‐M: 3	13 months	>= 50 point reduction in the IBS Symptom Severity Score	There was no significant difference in the primary endpoint achievement between the treatment group and the placebo group.
	9 (35%)	Single dose of 30 gm fecal suspension from patients' own stools			IBS‐D: 16, IBS‐M: 4			
El‐Salhy et al., 2020^2^	14 (26%)	Single dose of 30 gm donor feces mixed with 40 ml sterile saline	Gastroscope	17	IBS‐C: 20 IBS‐D: 22, IBS‐M: 13	18 months	>= 50 point reduction in the IBS Symptom Severity Score at 12 weeks	More patients in the active treatment groups showed inprovement in the IBS‐SSS than the placebo group. In addition, patients treated with 60 g of FMT showed more improvement in the scores than patients treated with 30 g of FMT.
	9 (16%)	Single dose of 60 gm donor feces mixed with 40 ml sterile saline		17	IBS‐C: 20 IBS‐D: 20, IBS‐M: 14			
	8 (15%)	Single dose of 30 gm patients' own feces mixed with 40 ml sterile saline		17	IBS‐C: 22 IBS‐D: 21, IBS‐M: 12			
Johnsen et al., 2020^25^	19 (35%)	Single dose of 50 ‐ 80 g of fresh faces homogenized in 200 mL of isotonic saline and 50 mL of 85% glycerol	Colonoscope	11.33	IBS‐D: 31, IBS‐M: 24	12 months	- The effect on QoL is significant at six months, but not maintained at twelve months. - The effect on fatigue is significant at six, but not at three and twelve months.	FMT induced significant relief in quality of life and fatigue.
	9 (32%)	Single dose of 50 ‐ 80 g of patients' own faces homogenized in 200 mL of isotonic saline and 50 mL of 85% glycerol		10.67	IBS‐D: 13, IBS‐M: 15			
Halkjær et al., 2019^19^	8 (32%)	Single daily dose of 25 capsules contining 50 gm frozen fecal matter for 12 days	Oral		IBS‐C: 7 IBS‐D: 7, IBS‐M: 11	6 months	>= 50 point reduction in the IBS Symptom Severity Score at 12 weeks	More patients in the placebo group showed improvement in the IBS‐SSS than the patients in the FMT group.
	8 (30.8%)	Single daily dose of 25 placebo capsules for 12 days			IBS‐C: 10 IBS‐D: 8, IBS‐M: 8			
Holster et al., 2019^26^	5 (62.5%)	Single dose of 150 ml FMT containing 30 g of freshly delivered feces mixed with sterile saline solution and 10% glycerol	Colonoscope	unknown: 0, 1–5 yr: 4, 5 yr: 4	IBS‐C: 1 IBS‐D: 5, IBS‐M: 2	6 months	>= 30 point decrease in gastrointestinal symptom rating scale‐IBS	There was no significant difference in the primary endpoint achievement between the active treatment group and the placebo group.
	3 (37.5%)	Single dose of 150 ml FMT containing 30 g of patients' own feces mixed with sterile saline solution and 10% glycerol		Unknown: 1, 1–5 yr: 3, 5 yr: 4	IBS‐C: 3 IBS‐D: 4, IBS‐M: 1			
Aroniadis et al., 2019^8^	16 (64%)	Single daily dose of 25 capsules contining 12 gm frozen fecal matter for 3 days	Oral	6.33	IBS‐D: 25	6 months	>= 50 point reduction in the IBS Symptom Severity Score at 12 weeks	There was no significant difference in the primary endpoint achievement between the active treatment group and the placebo group.
	14 (61%)	Single daily dose of 25 placebo capsules for 3 days		8.33	IBD‐D: 23			
Johnsen et al., 2018^27^	19 (35%)	Single dose of 50 ‐ 80 g of fresh faces homogenized in 200 mL of isotonic saline and 50 mL of 85% glycerol	Colonoscope	11.33	IBS‐D: 31, IBS‐M: 24	12 months	>= 75 point reduction in the IBS Symptom Severity Score at 12 weeks	More patients in the active treatment group showed improvement in the IBS‐SSS than the patients in the placebo group.
	9 (32%)	Single dose of 50 ‐ 80 g of patients' own faces homogenized in 200 mL of isotonic saline and 50 mL of 85% glycerol		10.67	IBS‐D: 13, IBS‐M: 15			

FMT: Fecal Microbiota transfer, IBS‐C: Irritable bowel syndrome constipation type, IBS‐D: Irritable bowel syndrome diarrhea type, IBS‐M: Irritable bowel syndrome mixed type.

**Figure 2:**
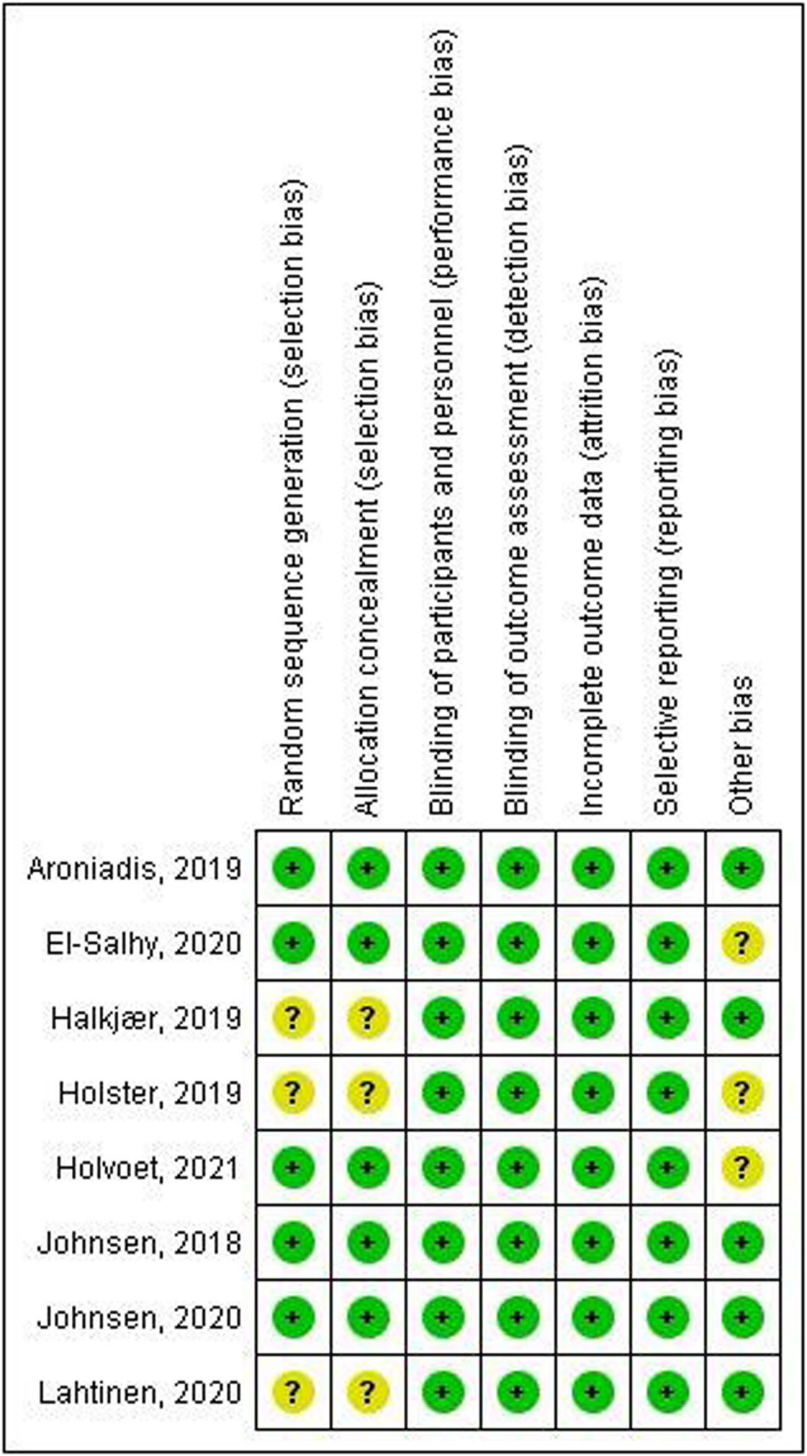
Risk of bias assessment

The risk of bias assessment revealed that the included studies were at low risk of bias. A summary of the risk of bias assessment domains is shown in Figure 2. A summary of the risk of bias assessment domains and authors' judgments with justifications are shown in Supporting Information: File 1.

**Figure 3:**
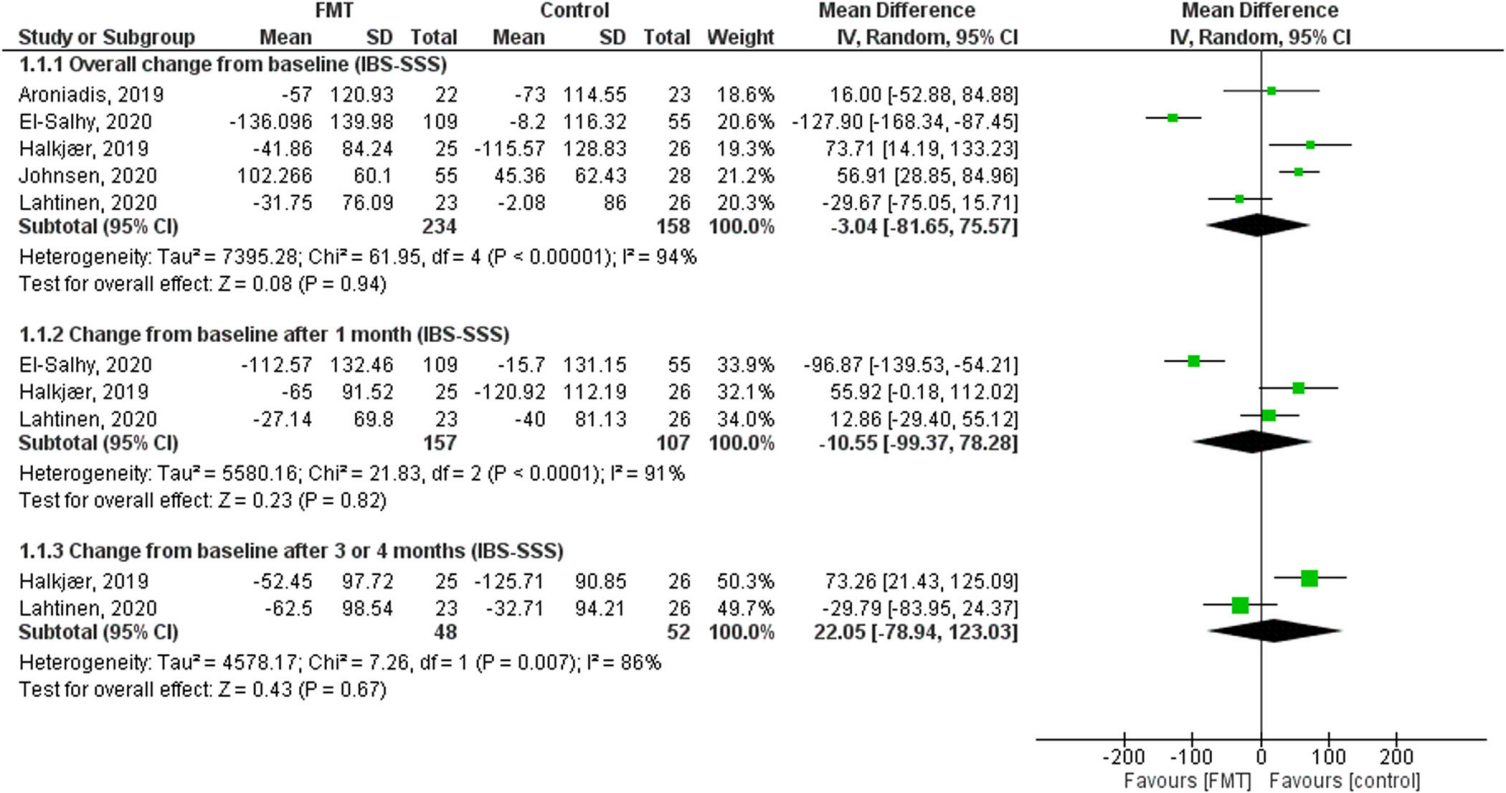
The results of IBS symptoms severity score (IBS‐SSS) outcomes

**Figure 4:**
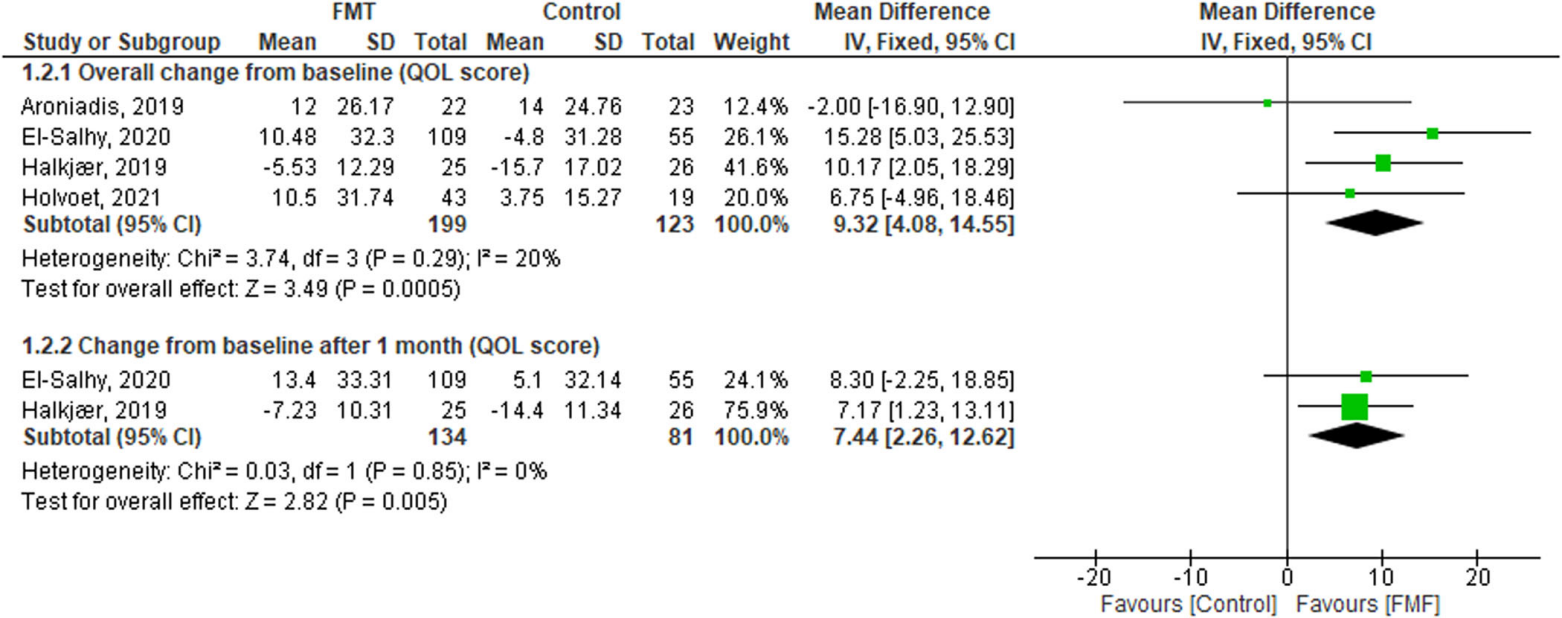
The results of quality of life (QOL) score outcomes

**Figure 5:**
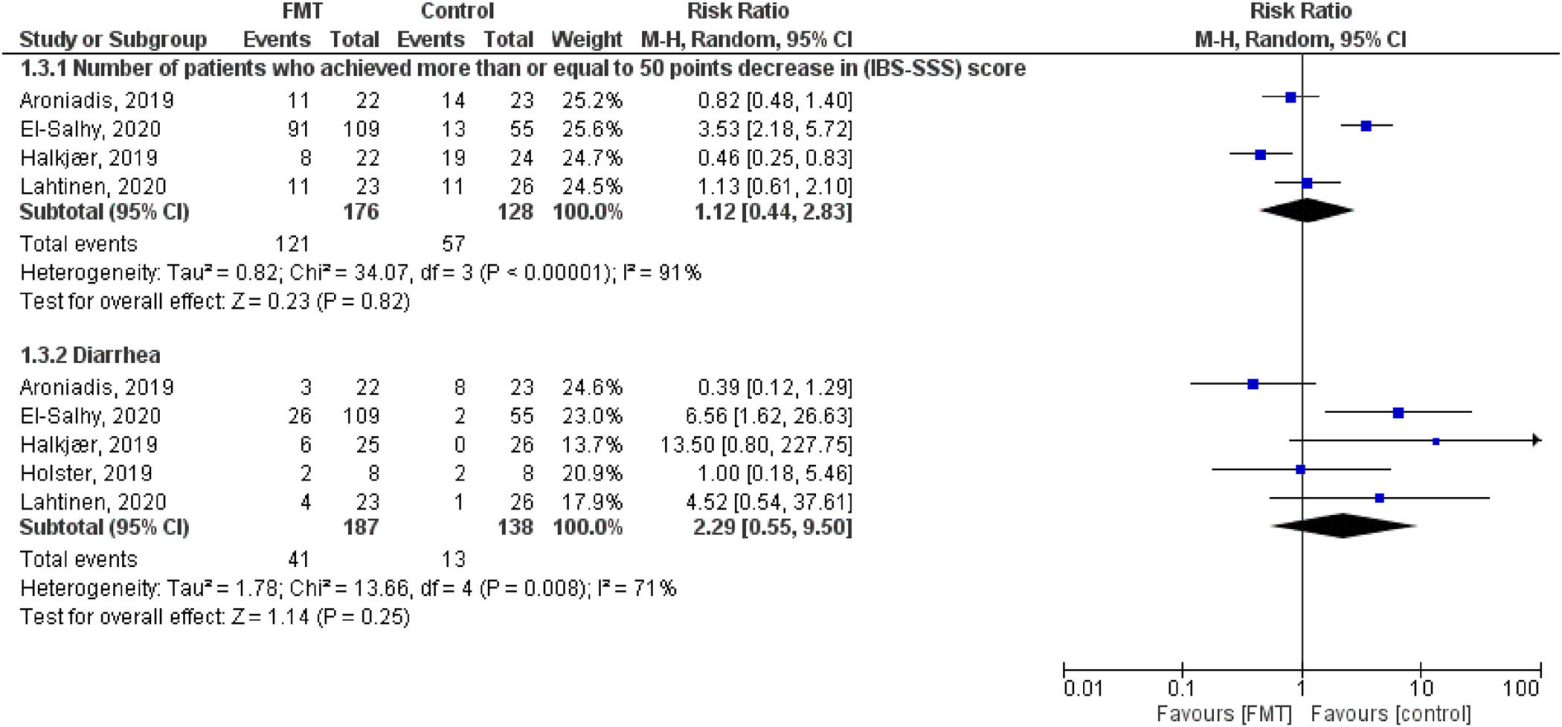
Forest plots of number of patients who achieved more than or equal to 50 points decrease in (IBS‐SSS) score and diarrhea

- Efficacy

(1) Overall change from the baseline in (IBS‐SSS).
  The pooled effect showed no statistically significant difference between the FMT and control groups (MD = −3.04, [95% CI = −81.65–75.57], *p* = 0.94). The observed heterogeneity was not solved by random effect and the leave one out test (*p* < 0.00001, *I*² = 94%) (Figure 3).
(2) Change from the baseline after 1 month (IBS‐SSS).
  The pooled effect showed no statistically significant difference between the FMT and control groups (MD = −10.55, [95% CI = −99.37–78.28], *p* = 0.82) (Figure 3). We observed heterogeneity (*p* < 0.0001, *I*² = 91%), so we performed leave one out test by removing El‐salhy et al.^2^ study and heterogeneity was resolved (*p* = 0.23, *I*² = 31%) and the effect estimate remained not significant (MD = 30.28, [95% CI = −11.14–71.70], *p* = 0.15).
(3) Change from the baseline after 3/4 months (IBS‐SSS).
  The pooled effect showed no statistically significant difference between the FMT and control groups (MD = 22.05, [95% CI = −78.94–123.03], *p* = 0.67) (Figure 3). The detected heterogeneity could not be resolved (*p* = 0.0007, *I*² = 86%).
(4) Overall change from the baseline in (QOL score).
  The pooled effect showed that FMT intervention significantly improves the QOL compared with the control groups (MD = 9.32, [95% CI = 4.08–14.55], *p* = 0.0005). We observed no significant heterogeneity among the studies (*p* = 0.29, *I*² = 20%) (Figure 4).
(5) Number of patients who achieved more than or equal to 50 points decrease in (IBS‐SSS) score.
  The pooled effect showed no statistically significant difference between the FMT and control groups (RR = 1.12, [95% CI = 0.44–2.83], *p* = 0.82). The observed heterogeneity was not solved by random effect and the leave one out test (*p* < 0.00001, *I*² = 91%) (Figure 5).
(6) Change from the baseline after 1 month (QOL score).
  The pooled effect showed that FMT intervention significantly improves QOL at 1 month compared to the control groups (MD = 7.044, [95% CI = 2.26–12.62], *p* = 0.005). We observed no significant heterogeneity between the two studies (*p* = 0.85, *I*² = 0%) (Figure 4).
(7) Number of respondents (Global improvement).
  The pooled effect showed no statistically significant difference between the FMT and control groups in all routes together (RR = 1.84, [95% CI = 0.82–2.65], *p* = 0.19) (Supporting Information: File 2, Figure 2).

We performed a subgroup analysis to test the effect of the type of route of administration on the number of respondents to treatment. The subgroup analysis was performed in oral, colonoscopy, nasojejunal, and gastroscope routes. The pooled effect showed no statistically significant difference between the FMT and control groups in colonoscopy and nasojejunal routes (RR = 0.77, [95% CI = 0.0.54–1.10], *p* = 0.16) (RR= 2.12, [95% CI = 0.95–4.71], *p* = 0.06), respectively (Supporting Information: File 2, Figure 2). The pooled effect showed a statistically significant association between the FMT and increased number of respondents in oral and gastroscope routes (RR = 2.03, [95% CI = 1.25–3.31], *p* = 0.004) (RR= 3.49, [95% CI = 2.47–4.94], *p* < 0.00001), respectively (Supporting Information: File 2, Figure 2).

**Figure 6:**
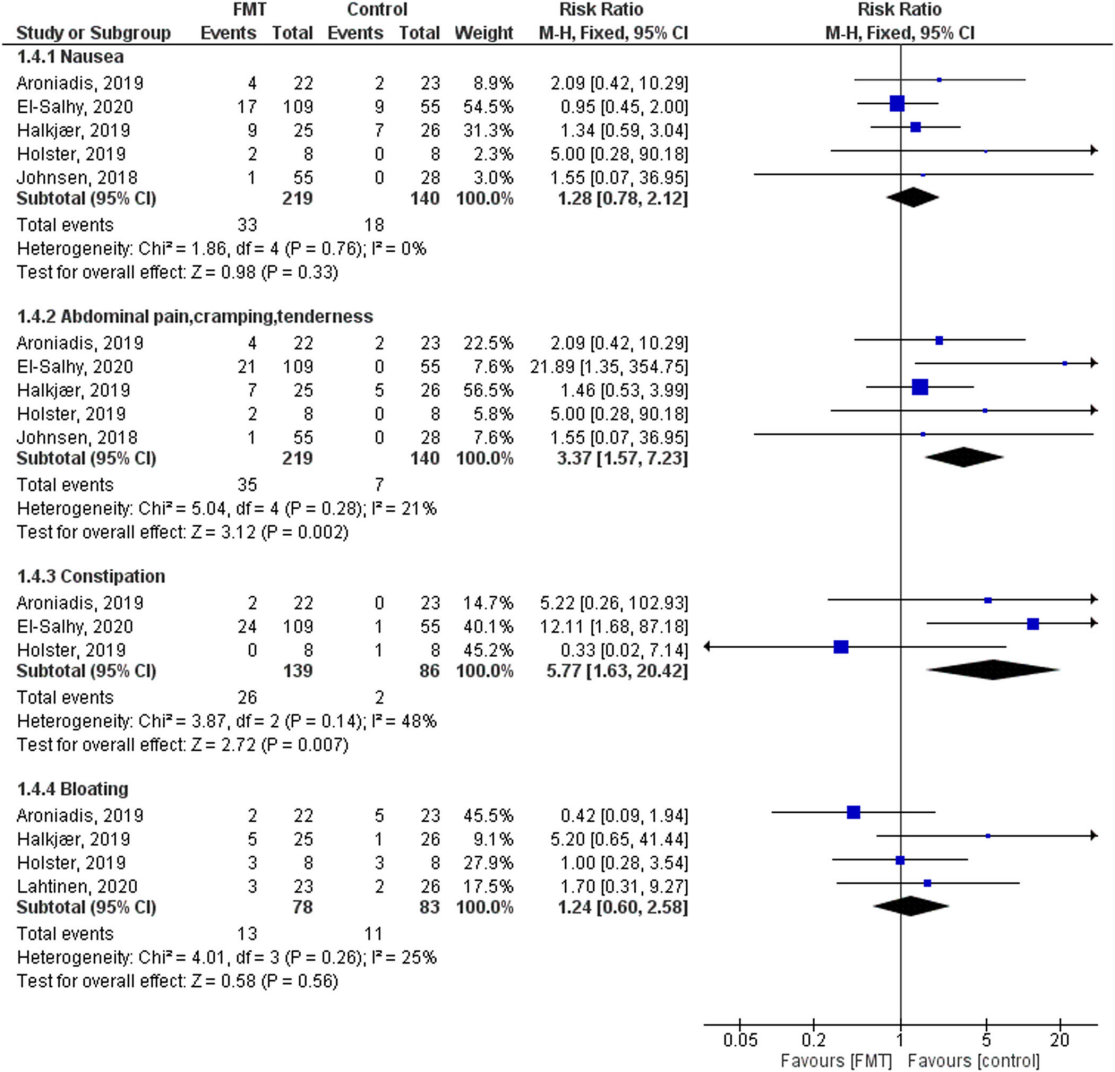
The difference in adverse events: nausea (abdominal pain, cramping, or tenderness), constipation, and bloating

- Adverse events

(1) Nausea
  The pooled results showed no statistically significant difference between the FMT and control groups (RR = 1.28, [95% CI = 0.78–2.12], *p* = 0.33). We observed no significant heterogeneity (*p* = 0.76, *I*² = 0%) (Figure 6).
(2) Abdominal pain/cramping/tenderness
  The pooled results showed that FMT is associated with more abdominal pain and cramping compared to the control group (RR = 3.73, [95% CI = 1.57–7.23], *p* = 0.002). We observed no significant heterogeneity (*p* = 0.28, *I*² = 21%) (Figure 6).
(3) Diarrhea
  The pooled effect showed no statistically significant difference between the FMT and control groups (RR = 2.29, [95% CI = 0.55–9.50], *p* = 0.25) (Figure 5). We observed heterogeneity (*p* = 0.008, *I*² = 71%), so we did leave one out test by removing Aroniadis et al.^8^ study and heterogeneity was solved (*p* = 0.25, *I*² = 26%)) and the results showed statistically significant difference between FMT and control groups (RR = 3.87, [95% CI = 1.29–11.59], *p* = 0.02).
(4) Constipation
  The pooled results showed that FMT is associated with more constipation compared to the control groups (RR = 5.77, [95% CI = 1.63–20.42], *p* = 0.007). We observed no significant heterogeneity (*p* = 0.14, *I*² = 48%) (Figure 6).
(5) Bloating
  The pooled effect showed no statistically significant difference between the FMT and control groups (RR = 1.24, [95% CI = 0.60–2.58], *p* = 0.56). We observed no significant heterogeneity (*p* = 0.26, *I*² = 25%) (Figure 6).

## 4 DISCUSSION

The results of our meta‐analysis showed that there is no statistically significant difference between the FMT group and the control group in the symptom severity score (IBS‐SSS) at 1, 3–4 months, and the overall change at the end of the study. NO statistically significant difference was found in the number of patients who achieved more than or equal to 50 points decrease in IBS‐SSS, and the number of respondents in the intervention group compared to the control group. The only outcome that shows a significant difference between the two groups is the QOL score, which indicates that (FMT) offers a better QOL for patients included in our study (MD = 9.32, 95% CI = 4.08–14.55, *p* = 0.0005). Based on the analysis of the adverse events, we found no statistically significant difference in nausea, diarrhea, and bloating. However, FMT was found to increase the risk of abdominal pain/cramping/tenderness, and constipation.

These results of the outcome (IBS‐SSS) agree with two of the included studies,^2^, ^8^ while contradicting two others,^25^, ^28^ in which, both show a significant improvement in symptoms in both groups. The observed improvement in the placebo group was more obvious in Johnsen et al.^25^ Lahtinen et al.^24^ observed a transient effect for the intervention and they referred it to the different donors used in the study and the unchanged faulty dietary habits of the recipients. On the other hand, all individual studies showed improvement in (QOL score) except Halkjær et al. which showed more improvement in the placebo group. Two of our included studies assessed the long‐term effect of FMT, and they both showed a decrease in the responders' percentage after 1 year to 55% and21%.^23^, ^24^ This indicates that the beneficial effect of FMT decays over time.

Our results dispute with the results of the previous meta‐analyses by Ianiro et al.^30^ and Xu et al.^29^ In the study by Xu et al.,^29^ they reported that a single dose introduced by colonoscopy and the nasojejunal tube is more effective than multiple oral doses. We did a subgroup analysis on both methods and found no significant improvement in IBS symptoms in either of them (Supporting Information: File 2, Figure 1). Similarly, in the study by Ianiro et al., they showed a significant improvement in the use of colonoscopy and nasojejunal tube. Interestingly, they found that oral placebo capsules were more significant than oral FMT.^30^ This conflict in results is mainly due to the different assessment methods used. They used dichotomous data for response or no response to FMT, which is of lower significance than SSS because it does not specify the different degrees of patients' responses. Xu et al.^29^ also included a study published as a conference abstract, which is of low quality of evidence and carries a higher risk of bias.

Also, we believe that our report provides better evidence compared to the recently published study by Wu et al.^31^ which showed conflicting results with ours. First, they performed an overall analysis of the adverse events related to FMT, which showed no significant increase compared to placebo, while we performed our analysis on each adverse effect separately and found a significant increase in abdominal pain and constipation in FMT compared to placebo. They combined the adverse events in one outcome, which is misleading because adverse events differ in degree of significance and severity. Second, after a subgroup analysis on the route of administration of FMT, they reported that colonoscopy was associated with a more improvement in the global symptoms of IBS compared to placebo, while the oral route was inferior to placebo. We used the IBS‐SSS, which is a more reliable assessment score than the global symptoms score, in our subgroup analysis and found that the oral route is associated with more improvement compared to placebo, while colonoscopy was inferior to placebo. Third, we included more clinical trials, which further validates our results.

Based on the variation in the GIT bacterial flora of IBS patients from the normal population and the analysis of the specific bacteria incriminated in each subtype,^32^ several treatment modalities in addition to FMT were developed to target and modulate the bacterial flora of IBS patients. Probiotics are an effective treatment, which aims at restoring the natural balance of GIT flora by increasing certain species of beneficial bacteria.^33^ Several types of bacteria were used as probiotics. Bacillus Coagulans MTCC 5856 is a very effective bacterial strain in reducing bloating and abdominal pain in diarrheal dominant IBS patients.^34^ It outstands other strains by its great durability for heat and acidic nature in the GIT due to its spore‐forming nature, thus, it can have a longer duration of action.^35^ Future therapies for modulating GIT flora are now under development. A new technique using bacteriophages to target specific intestinal bacteria is now approved by FDA for further research.

Another promising therapy is the stem cell‐based gut‐on‐a‐chip. It creates a microenvironment for testing potential therapies and customizing them for each patient.^36^ Using antibiotics that are poorly absorbed from GIT like rifaximin and neomycin is now used more widely, but its main flaw is the lack of specificity as it may affect the harmless flora as well.^37^ Now, precision antimicrobial peptides called selectively targeted antimicrobial peptides were developed to target certain species without affecting the normal flora. This therapy was only used in dental caries, but in the future, it can be effective in IBS as well.^38^ Another similar technique is using the contractile nanotubes produced by certain bacteria that can attach to certain receptors on the cell wall of other bacteria and kill them. We can target certain pathogenic bacteria by modulating those contractile tubes to make them attach to the surface receptor of the pathogenic bacteria.^39^

Diet is another important modifiable element in the pathogenesis of IBS. The two main proven dietary plans are eating low fermentable oligosaccharides, disaccharides, monosaccharides and polyols (FODMAP) and gluten‐free food.^40^ FODMAPs are short‐chain carbohydrates that are poorly absorbed from the intestinal lumen. They have highly osmotic power, so they increase bloating and abdominal pain. They are also easily fermentable by intestinal flora with gas production which increases the feeling of boating.^41^ A low FODMAP diet was shown to reduce IBS symptoms by 68% and offers a better QOL.^42^ On the other hand, gluten induces IBS symptoms without patients actually having celiac disease. This condition is called “non‐celiac gluten sensitivity (NCGS).”^43^ Gluten‐free diet is mainly effective in the diarrheal subtype where it is shown to reduce stool frequency in patients who are HLA‐DQ2/8‐positive.^44^ The mechanism is still unclear but it may be due to a genetically determined immune response.

For better symptom improvement in IBS patients, therapeutic measures and diet modification should be part of a more comprehensive management plan. The three main pillars for management are medications, dietary plans, and behavioral therapy.^45^ There is a reciprocal relationship between brain and gut mediated by hormones, CNS, and PNS. Chronic Stress, fear of symptoms, and lack of control of disease exacerbate the patient's symptoms and decrease their QOL.^46^ Therefore, different behavioral therapies are now used and proved great efficacy in decreasing patients' symptoms and increasing their QOL such as gastrointestinal‐focused cognitive behavioral therapy and gut‐directed hypnotherapy.^47^ This requires a multidisciplinary team of gastroenterologists, dietitians, gut‐focused hypnotherapists, psychiatrists, and cognitive‐behavioral physiotherapists.^48^ Telephone and web‐based cognitive behavioral therapy are showing even better efficacy than standard treatment but are much more cost‐effective, yet long‐term efficacy is not well established.^49^

Three studies^24^, ^26^, ^28^ were limited by the small sample size of the patients included in both groups. Four other studies^2^, ^19^, ^24^, ^27^ did not specify the type of the IBS major symptoms and this heterogeneity may affect the patients' response variably. Mixed donors for FMT, who have different microbiota compositions is another limitation noticed in three other studies.^19^, ^25^, ^27^

The main strength points of our study are as follows: first, we are the first meta‐analysis to plot the degree of improvement in symptoms by using symptom severity score as a scaling system, unlike the previous meta‐analyses which plotted the improvement of patients as dichotomous data that does not show the degree of improvement in these patients. Second, we included RCT only and unlike the previous meta‐analysis, we did not include single‐arm trials and conference abstract, which increases the impact of our study. Third, we did a precise screening for all databases present and included all eligible studies. We also assessed the risk of bias for all included studies and it was generally low, which increases the quality of evidence in our study. However, our study was limited by the significant heterogeneity found in most of the results outcomes and that heterogeneity mostly could not be resolved by the normal statistical ways, which implies that our results are not biologically plausible. Our systematic review was not registered. However, we described our methodology precisely and provided a PRISMA checklist and justified the authors' assessment of the risk of bias. Moreover, we could not analyze some of the outcomes because they were not assessed in all the included trials.

In conclusion, FMT is not an effective treatment for IBS symptoms whether it is administered orally, by colonoscopy, gastroscopy, or through a nasojejunal tube. Although it may show a transient effect in some patients, this effect wears off drastically over time, and even after repeated administration, it does not show the initial effect, which suggests that it is only a placebo effect. Future studies should be directed toward probiotics and newer technologies in modulating GIT bacterial composition, as well as diet modification. Integrated management for IBS patients is now strongly advised as it addresses all pathological aspects of the disease.

## AUTHOR CONTRIBUTIONS

**Yomna Ali Abdelghafar**: Conceptualization; data curation; writing – original draft; writing – review & editing. **Yossef Hassan AbdelQadir**: Conceptualization; formal analysis; methodology; software; supervision; writing – review & editing. **Karam R. Motawea**: Formal analysis; visualization. **Sara Amr Nasr**: Conceptualization; data curation; validation. **Hoda Aly Mohamed Omran**: Data curation; validation. **Mohamed Mohamed Belal**: Data curation; validation. **Mohamed Mahdy Elhashash**: Data curation; validation. **Ahmed Alaa AbdelAzim**: Formal analysis; visualization. **Jaffer Shah**: Formal analysis; visualization.

## CONFLICT OF INTEREST

The authors declare no conflict of interest.

## TRANSPARENCY STATEMENT

The lead author Jaffer Shah affirms that this manuscript is an honest, accurate, and transparent account of the study being reported; that no important aspects of the study have been omitted; and that any discrepancies from the study as planned (and, if relevant, registered) have been explained.

## Supporting information

Supporting information.

Supporting information.

## Data Availability

Data sharing is not applicable because no new data was generated except for the data presented in the results section of the manuscript.
