# Supplementary material for: Efficacy and safety of fecal microbiota transplant in irritable bowel syndrome: An update based on meta‐analysis of randomized control trials
Source: Health Sci Rep. 2022 Sep 12;5(5):e814. doi: 10.1002/hsr2.814 (PMC9466358; doi:10.1002/hsr2.814)
Supplement: Supplementary file 1 — Supporting information. [file HSR2-5-e814-s002.docx]

**
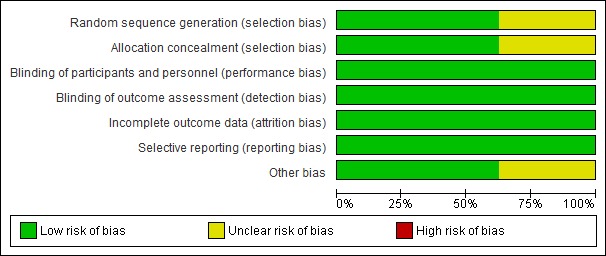
**

**Aroniadis, 2019**

**Risk of bias table**

| **Bias** | **Authors' judgement** | **Support for judgement** |
| --- | --- | --- |
| Random sequence generation (selection bias) |  | A randomisation scheme with a block size of four was computer generated |
| Allocation concealment (selection bias) |  | An independent staff member assigned the treatments according to consecutive numbers that were kept in sealed opaque envelopes. |
| Blinding of participants and personnel (performance bias) |  | Both participants and all members of the study teams at each site were masked to the computer-generated randomisation scheme.  Double blinding was achieved by the preparation of identical appearing capsules |
| Blinding of outcome assessment (detection bias) |  | Both participants and all members of the study teams at each site were masked to the computer-generated randomisation scheme.  Double blinding was achieved by the preparation of identical appearing capsules |
| Incomplete outcome data (attrition bias) |  | Analyses were done according to the intention-to-treat principle |
| Selective reporting (reporting bias) |  | The study was registered and protocol matches final published |
| Other bias (Fund mainly and conflict of interest) |  | The funder of the study had no role in study design, data collection, data analysis, data interpretation, or the writing of the report. All authors had access to the study data and reviewed and approved the final manuscript. |

**El-Salhy, 2020**

**Risk of bias table**

| **Bias** | **Authors' judgement** | **Support for judgement** |
| --- | --- | --- |
| Random sequence generation (selection bias) |  | using a Web-based system (http://www. randomization. com) by a nurse who was not involved in the trial |
| Allocation concealment (selection bias) |  | The randomisation key was revealed to the researcher and patients after the trial had ended |
| Blinding of participants and personnel (performance bias) |  | The patients and researchers involved in the study were blinded to the randomisation.  The researcher who prepared the transplant was not aware of the identity of the faecal sample used for transplantation. |
| Blinding of outcome assessment (detection bias) |  | The patients and researchers involved in the study were blinded to the randomisation.  The researcher who prepared the transplant was not aware of the identity of the faecal sample used for transplantation. |
| Incomplete outcome data (attrition bias) |  |  |
| Selective reporting (reporting bias) |  | The study was registered and protocol matches final published |
| Other bias (Fund mainly and conflict of interest) |  | Data are available upon reasonable request |

**Halkjær, 2019**

**Risk of bias table**

| **Bias** | **Authors' judgement** | **Support for judgement** |
| --- | --- | --- |
| Random sequence generation (selection bias) |  | The randomisation was done in blocks of 4 by a researcher, not involved in the patients’ treatment and was generated by using the website Randomization. com |
| Allocation concealment (selection bias) |  | . |
| Blinding of participants and personnel (performance bias) |  | Investigators, patients and outcome assessors were kept masked to the allocation and intervention. The randomisation key was revealed to the researchers when participants completed the 6-month follow-up and data analysis was completed. |
| Blinding of outcome assessment (detection bias) |  | Investigators, patients and outcome assessors were kept masked to the allocation and intervention. The randomisation key was revealed to the researchers when participants completed the 6-month follow-up and data analysis was completed. |
| Incomplete outcome data (attrition bias) |  | intention to treat analysis |
| Selective reporting (reporting bias) |  |  |
| Other bias (Fund mainly and conflict of interest) |  | The funding sources had no role in the study design, data collection, interpretation of analysis, writing of the manuscript or decision to submit the publication. |

**Holster, 2019**

**Risk of bias table**

| **Bias** | **Authors' judgement** | **Support for judgement** |
| --- | --- | --- |
| Random sequence generation (selection bias) |  | no definitive method detected "The randomization list was generated by a researcher not involved in the study." |
| Allocation concealment (selection bias) |  | . |
| Blinding of participants and personnel (performance bias) |  | All participants and investigators remained blinded until the analyses of the primary outcome and the symptom scale data were completed. Statistical analysis of all the symptom scale data was performed blinded |
| Blinding of outcome assessment (detection bias) |  | All participants and investigators remained blinded until the analyses of the primary outcome and the symptom scale data were completed. Statistical analysis of all the symptom scale data was performed blinded |
| Incomplete outcome data (attrition bias) |  |  |
| Selective reporting (reporting bias) |  | The study was registered and protocol matches final published |
| Other bias (Fund mainly and conflict of interest) |  | . |

**Holvoet, 2021**

**Risk of bias table**

| **Bias** | **Authors' judgement** | **Support for judgement** |
| --- | --- | --- |
| Random sequence generation (selection bias) |  | by personnel not involved in the clinical assessment using a randomization website. |
| Allocation concealment (selection bias) |  | Non-study personnel performed the final allocation, standardizing the active and placebo transplants to make them identical in appearance and temperature. Study participants and investigators were blinded to treatment allocation. |
| Blinding of participants and personnel (performance bias) |  | Study participants and investigators were blinded to treatment allocation. |
| Blinding of outcome assessment (detection bias) |  | Study participants and investigators were blinded to treatment allocation. |
| Incomplete outcome data (attrition bias) |  | Data were analyzed per the intention-to-treat protocol. |
| Selective reporting (reporting bias) |  | The study was registered and protocol matches final published |
| Other bias (Fund mainly and conflict of interest) |  | . |

**Johnsen, 2018**

**Risk of bias table**

| **Bias** | **Authors' judgement** | **Support for judgement** |
| --- | --- | --- |
| Random sequence generation (selection bias) |  | A researcher, not involved in the trial, created the allocation sequence using a randomisation website. |
| Allocation concealment (selection bias) |  | This randomisation sequence was sealed in opaque envelopes |
| Blinding of participants and personnel (performance bias) |  | Otherwise, patients, investigators, and outcome assessors were kept masked to the allocation and intervention. |
| Blinding of outcome assessment (detection bias) |  | To maintain blinding, an independent research group from the University of Oslo did an interim analysis of efficacy and safety |
| Incomplete outcome data (attrition bias) |  |  |
| Selective reporting (reporting bias) |  | The study was registered and protocol matches final published |
| Other bias (Fund mainly and conflict of interest) |  | The funder of the study had no role in the study design, data collection, data analysis, data interpretation, or writing of the report. |

**Johnsen, 2020**

**Risk of bias table**

| **Bias** | **Authors' judgement** | **Support for judgement** |
| --- | --- | --- |
| Random sequence generation (selection bias) |  | A researcher, not involved in the trial, created the allocation sequence using a randomisation website. |
| Allocation concealment (selection bias) |  | This randomisation sequence was sealed in opaque envelopes |
| Blinding of participants and personnel (performance bias) |  | Otherwise, patients, investigators, and outcome assessors were kept masked to the allocation and intervention. |
| Blinding of outcome assessment (detection bias) |  | To maintain blinding, an independent research group from the University of Oslo did an interim analysis of efficacy and safety |
| Incomplete outcome data (attrition bias) |  |  |
| Selective reporting (reporting bias) |  | The study was registered and protocol matches final published |
| Other bias (Fund mainly and conflict of interest) |  | The funder of the study had no role in the study design, data collection, data analysis, data interpretation, or writing of the report. |

**Lahtinen, 2020**

**Risk of bias table**

| **Bias** | **Authors' judgement** | **Support for judgement** |
| --- | --- | --- |
| Random sequence generation (selection bias) |  | The randomisation was done in blocks of six by a study nurse who was not involved in the treatment of the patients. |
| Allocation concealment (selection bias) |  | . |
| Blinding of participants and personnel (performance bias) |  | Decoding was only performed when all the patients had completed the 52-week follow-up. |
| Blinding of outcome assessment (detection bias) |  | Decoding was only performed when all the patients had completed the 52-week follow-up. |
| Incomplete outcome data (attrition bias) |  |  |
| Selective reporting (reporting bias) |  | The study was registered and protocol matches final published |
| Other bias (Fund mainly and conflict of interest) |  | The research was independent of the funding sources. |

**Footnotes**
