# Supplementary material for: Efficacy and safety of fecal microbiota transplant in irritable bowel syndrome: An update based on meta‐analysis of randomized control trials
Source: Health Sci Rep. 2022 Sep 12;5(5):e814. doi: 10.1002/hsr2.814 (PMC9466358; doi:10.1002/hsr2.814)

- **Subgroup analysis of the IBS-SSS based on the route of administration.**

We did subgroup analysis to test the effect of the type of route of administration on the overall change from the baseline in (IBS-SSS). The subgroup analysis was performed between colonoscopy and oral routes. The pooled effect showed no statistically significant difference between the FMT and control groups in both colonoscopy and oral routes (MD= 15.53, [95% CI= -69.23 to 100.29], *P* = 0.72), (MD= 47.57, [95% CI= -8.74 to 103.87], *P* = 0.10), respectively (figure 3).

Figure1. Subgroup analysis based on route of administration (overall change from the baseline in (IBS-SSS).


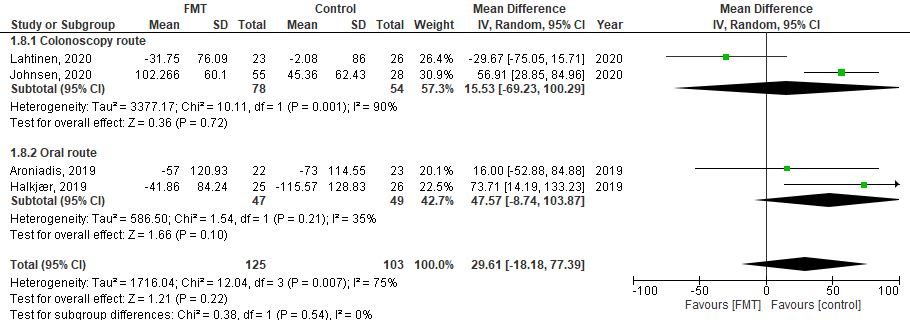


- **Number of respondents and subgroup analysis basedon the route of administration.**

We did subgroup analysis to test the effect of the type of route of administration on the number of respondents to treatment. The subgroup analysis was performed in oral, colonoscopy, nasojejunal and gastroscope routes. The pooled effect showed no statistically significant difference between the FMT and control groups in colonoscopy and nasojejunal routes (RR= 0.77, [95% CI= 0.0.54 to 1.10], *P* = 0.16), (RR= 2.12, [95% CI= 0.95 to 4.71], *P* = 0.06), respectively (figure 2). The pooled effect showed a statistically significant association between the FMT and increased number of respondents in oral and gastroscope routes (RR= 2.03, [95% CI= 1.25 to 3.31], *P* = 0.004), (RR= 3.49, [95% CI= 2.47 to 4.94], *P* < 0.00001), respectively (figure 2). The pooled effect showed no statistically significant difference between the FMT and control groups in all routes together (RR= 1.84, [95% CI= 0.82 to 2.65], *P* = 0.19) (figure 2).

Figure2. Subgroup analysis based on route of administration (number of respondents to treatment).


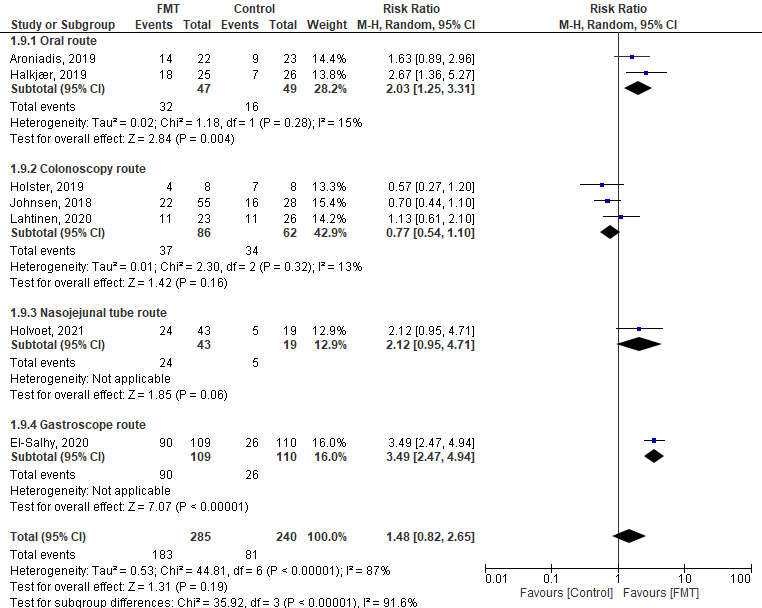

Supplement: Supplementary file 2 — Supporting information. [file HSR2-5-e814-s001.docx]
